# A novel concealed posterior axillary approach for scapula glenoid fractures – a multicenter clinical study

**DOI:** 10.1097/JS9.0000000000002971

**Published:** 2025-07-17

**Authors:** Qingyu Wang, Yong Xing, Jian Ding, Dawei Yang, Kaifa You, Kun Wang, Yun Tian, Dankai Wu

**Affiliations:** a Department of Traumatology, The Second Hospital of Jilin University, Changchun, P. R. China; b Department of Orthopaedics, The Third Hospital of Peking University, Beijing, P. R. China; c Department of Orthopaedic Surgery, Shanghai Sixth People’s Hospital Affiliated to Shanghai Jiao Tong University School of Medicine, Shanghai, P. R. China; d Department of Orthopaedic, The Fourth Affiliated Hospital of Harbin Medical University, Harbin, P. R. China; e Department of Orthopaedics, Shanghang County Hospital, Fujian, P. R. China; f Department of Upper Limb Orthopaedics, Zhengzhou Orthopaedic Hospital, Zhengzhou, P. R. China

**Keywords:** axillary approach, Bankart lesions, multicenter clinical study, scapula glenoid fracture

## Abstract

Glenoid fractures of the scapula can be treated via anterior deltopectoral, Judet, modified Judet, or posterior minimally invasive approaches; however, these may result in soft tissue injury, hematoma formation, nerve damage, or inadequate internal fixation stability. While arthroscopy minimizes soft tissue dissection, it may pose challenges in visualizing inferior glenoid fragments. To address these potential complications, we designed a new posterior axillary approach, the Tian Yun–Wu Dankai approach (T-W approach), in the treatment of certain glenoid fractures of scapula. We retrospectively reviewed 101 patients (Iderberg Ia, 71; II, 24; IV, 1; and V, 5) treated for glenoid fractures of the scapula using the T-W approach across six medical centers. All patients were followed-up for at least 12 months (range, 12–36 months). Postoperative radiographs and computed tomography scans indicated satisfactory fracture reduction and rigid fixation with plates and screws. The mean incision length, blood loss, and operation time were 10.8 ± 1.3 cm, 104.0 ± 32.7 mL, and 98.0 ± 32.0 min, respectively. At the last follow-up, the mean DASH and Constant scores were 14.0 ± 5.1 and 88.6 ± 3.7, respectively. The mean range of motion of forward flection, abduction, and external rotation was 160.0 ± 19.0°, 156.0 ± 20.0°, and 64.0 ± 8.5°, respectively. Two patients experienced delayed incision healing that resolved with dressing changes. Mild heterotopic ossification was observed in two patients; however, it did not affect shoulder function. Postoperative traumatic shoulder arthritis was observed in one patient. Three patients experienced numbness in the surgical area posterior to the incision site. The T-W approach to treat scapular glenoid fractures (all Ideberg Ia and II, parts IV and V) can fill the gap in the non-inferior approach for the shoulder joint. This approach offers several advantages, including minimal tissue damage, short operative time, effective reduction, rigid fixation, and high functional scores with aesthetic benefits.

## Introduction

Glenoid fractures (10% of scapular fractures) often involve instability or dislocation^[1,2]^. Although the deltopectoral approach is commonly used to treat anterior glenoid fractures, it requires incision of the subscapularis muscle and capsule. The Judet approach is effective for internal fixation of scapular fractures but requires dissection of the teres minor and infraspinatus muscles, causing significant soft tissue trauma^[3]^. In 2004, Obremskey *et al*^[4]^ introduced a modification to the conventional Judet approach. This novel technique preserved the integrity of the teres minor, infraspinatus, suprascapular nerve, and axillary nerve while providing adequate exposure to all bony structures. However, a large skin flap may cause complications such as bleeding, seroma formation, or nerve injury. In 2018, Jones *et al*^[5]^ reported that approaching between the teres major and teres minor in the treatment of scapular neck fractures effectively avoided deltoid dissection. However, postoperative complications such as weakened external rotation of the upper limb, brachial plexus paralysis, and radial nerve injury occurred.

We designed a novel posterior axillary approach (T-W approach) for the treatment of some types of glenoid fractures (all Ideberg Ia and II, parts IV and V). This approach offers significant advantages in exposing the anterior inferior, inferior, and partial posterior margins of the glenoid fossa and the neck and lateral borders of the scapula. The incision was small and concealed, without rotator cuff tissue resection. We summarized and analyzed the advantages and disadvantages of this new approach and its initial clinical results. This approach was eponymously designed to acknowledge the foundational work of Prof. Tian Yun and Prof. Wu Dankai, characterizing the posterior axillary approach. In accordance with the TITAN guidelines^[6]^ for transparent reporting, no artificial intelligence tools were used in the conception, design, or execution of this study.

### Patients and methods

This multicenter retrospective study (six Chinese hospitals, April 2018–June 2022) included 101 patients (66 male and 35 female) with scapular glenoid fractures requiring open reduction/internal fixation^[7]^. Inclusion criteria were age ≥18 years and no open wounds at incision sites. The exclusion criteria included a follow-up of <12 months, preoperative delay of >4 weeks, or surgical area infections. The T-W approach was systematically employed for all consecutive patients meeting the following criteria: (1) Ideberg Ia fractures occurring between 1:00 and 6:00 (fixation range from 3:00 to 7:00); and (2) all Ideberg II fractures and certain parts of Ideberg IV and V fractures, where the fracture lines were located on the lower side of the glenoid. A combined medial approach may be required for scapular fractures involving medial edges. Approach selection was solely based on fracture pattern suitability, as determined by the attending trauma surgeon, without patient involvement. Ethics approval was obtained from all participating institutions.

### Surgical procedure

**Figure 1.**
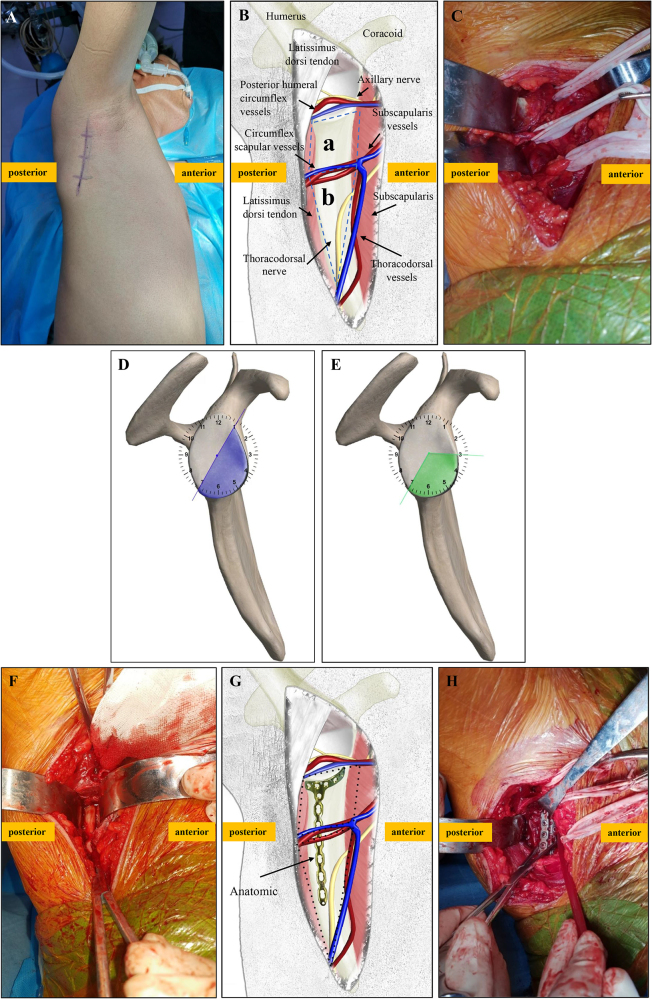
Illustrations depicting the T-W approach. (A) The patient is shown in the lateral decubitus position and the incision is marked on the body. (B) The anatomical structure of the “upper quadrilateral space (a)” and “lower trilateral space (b).” (C) The axillary nerve, circumflex scapular vessels, and thoracodorsal vessels are located and separated. (D) The exposure of the glenoid cavity ranges from 1:00 to 7:00. (E) The fixation of the glenoid cavity ranges from 3:00 to 7:00. (F) The fracture is exposed and reduced under direct vision. (G) Schematic diagram of the T-W approach combined with an anatomic plate. (H) The fracture is reduced and fixed with one or two plates.

**Figure 2.**
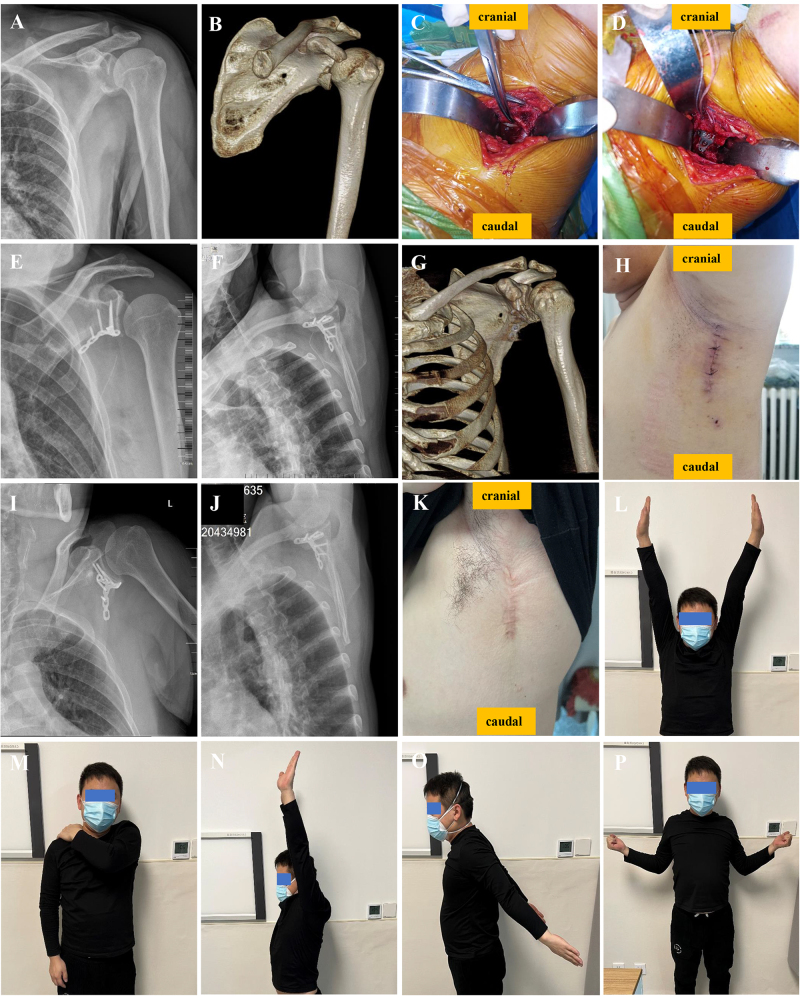
The technique and outcomes associated with the T-W approach for an Ideberg Ia fracture. (A and B) Preoperative radiography and 3D-CT assessments. (C and D) Intraoperative photograph showing the fracture exposed and fixed with one plate. (E–H) Postoperative radiograph, 3D-CT image, and incision. (I–P) Radiograph, incision, and shoulder joint activity 14 months postoperatively.

The patient was placed in a lateral position with the arm abducted at 90–120°. The incision was marked along the posterior axillary line (Fig. 1A). The skin and superficial and deep fasciae were incised, and the tendon of the latissimus dorsi was exposed at the posterior edge of the incision. The operating area is divided into the “upper quadrilateral foramen” and the “lower trilateral foramen” with circumflex scapular vessels, which are perpendicular to the scapula (Fig. 1B). The axillary nerve was located 1.0 cm from the glenoid rim and required protection during surgery (Fig. 1C). However, experienced surgeons do not require exposure of the axillary nerve during this procedure. The T-W approach enabled exposure of the glenoid cavity from approximately 1:00 to 7:00 (Fig. 1D), with a fixation range from 3:00 to 7:00 (Fig. 1E). The fracture was exposed, reduced (Fig. 1F) and temporarily stabilized using 1.5 mm K-wires under direct vision through the “upper quadrilateral foramen” and/or the “lower trilateral foramen.” Anatomical or T-shaped plates were placed along the lateral border of the scapula (Fig. 1G and H). At the proximal end of the plate, screws were placed parallel to the tangential line of the glenoid and perpendicular to the fracture line. Two or three screws were added at the distal end to form a buttress plate fixation (Fig. 2). Smaller fragments can be stabilized using either a miniplate or an anchor. Larger fragments, particularly those affecting the neck or body of the scapula, can be stabilized using anatomical plate or 2.7 mm reconstruction plate (Supplementary Digital Content, Figure 1, available at: http://links.lww.com/JS9/E778). Occasionally, two plates (lateral and anterolateral) are used to enhance fixation in a stereo-fixed manner. Glenoid and scapular body fractures can be managed using a combined medial border approach, which exposes the fractures between the infraspinatus and rhomboid muscles (Supplementary Digital Content, Figure 2, available at: http://links.lww.com/JS9/E778). Notably, when the joint capsule is incised, and the humeral head is pushed aside, the entire articular surface of the glenoid can be fully exposed via the T-W approach. Conversely, without an incision in the joint capsule, anterior and/or inferior glenoid, neck, and transverse scapular body fractures can be accessed, reduced, and fixed extra-articular using the T-W approach. The reduction accuracy can be verified via C-arm fluoroscopy and direct palpation when needed.

**HIGHLIGHTS**

- T-W approach offers minimal tissue damage and aesthetic benefits.
- 101 glenoid fracture cases showed good reduction and fixation.
- High postoperative shoulder function and low complication rate.
- T-W approach fills a gap in non-inferior glenoid fracture approaches.

Active/passive exercises (scapular squeezing and pendulum) were initiated postoperative day 2. This involved scapular squeezing, pendulum, and wall-climbing exercises. Full range of motion (ROM) and strengthening exercises commenced at 4 weeks. All restrictions were lifted after 3 months.

Follow-ups at 1.5–36 months assessed fracture healing^[8]^, complications, and functional scores (disability of the shoulder, arm, and hand questionnaire [DASH]^[9]^ and constant scores^[10]^). All statistical analyses were performed.

## Results

A total of 101 patients (mean age: 53.2 ± 13.9 years) were followed for 17.8 ± 6.6 months. The fractures were classified according to Ideberg^[11]^ and the causes of injury were fall (77 cases), traffic accidents (20 cases), and crushing (4 cases) (Table 1; Supplementary Digital Content, Figure 3, available at: http://links.lww.com/JS9/E778). The average preoperative time was 5.6 ± 3.8 days. The T-W approach achieved minimal invasiveness (incision: 10.8 ± 1.3 cm; blood loss: 104.0 ± 32.7 mL; operative time: 98.0 ± 32.0 min) and near-anatomical reduction (100% union). Functional outcomes were excellent (DASH: 14.0 ± 5.1; Constant: 88.6 ± 3.7; ROM: flexion 160.0 ± 19.0°, abduction 156.0 ± 20.0°, external rotation 64.0 ± 8.5°; Supplementary Digital Content, Table, available at: http://links.lww.com/JS9/E779). Complications included delayed wound healing (two resolved with dressings), mild heterotopic ossification (two, no functional impact), transient numbness (three; two recovered fully at 2 years), and one case of non-disabling traumatic arthritis. All incisions healed primarily except two delayed cases.

**Table 1:** Patient characteristics

Variable	Number of cases
Mean age ± SD (yr)	53.2 ± 13.9
Sex (no. [%])	
Male	66 (65.3)
Female	35 (34.7)
Causes of injury (no. [%])	
Fall	77 (76.2)
Traffic	20 (19.8)
Crushing	4 (4.0)
Iderberg classification (no. [%])	
Ia	71(70.30)
II	24(23.76)
IV	1(0.99)
V	5(4.95)

SD, standard deviation.

## Discussion

In this study, all patients were treated using the T-W approach and favorable outcomes were achieved without cutting off any tendon or stripping any muscles. While anatomical studies suggest theoretical applicability to Ideberg Ib fractures between 6:00 and 7:00, clinical validation requires further investigation. No Ib fractures meeting these criteria were encountered during the study period.

In 1964, Judet introduced a posterior approach for the treatment of scapular fractures^[3]^. This method requires dissection of the teres minor and infraspinatus muscles. A modification of the traditional Judet approach, reported by Brodsky *et al* in 1987, provides good access to the lateral border by utilizing the interval between the teres minor and teres major^[12]^. In 1993, Wirth *et al*^[13]^ described a method of splitting the deltoid muscle in the posterior approach with excellent exposure and no complications, and preserved the strength and function of the posterior deltoid. Gauger *et al*^[14]^ reported that incisions could be made along the anatomical bony perimeter to access the scapular borders (scapular body, neck, and posterior glenoid). Although these approaches provide satisfactory exposure for most scapular fractures, complications, such as hematoma, hemorrhage, nerve damage, heterotopic ossification, and impaired shoulder function may occur^[15,16]^. In the posterior approach, exposure and fixation are not feasible if the bone fragment is positioned anterior or inferior to the glenoid cavity. Conversely, this can be achieved using the T-W approach.

The T-W approach has several advantages: (1) It avoids muscle dissection (vs. Judet^[3]^, anterior^[17,18]^, or posterior^[12^] [^13]^ approaches), preserves rotator cuff integrity, and uses intermuscular spaces. In contrast to arthroscopy, which is limited to small anterior fractures (<25% glenoid involvement^[11,19]^) and cannot address scapular body fractures^[20]^ the T-W approach enables rigid fixation of broader fracture patterns while minimizing soft tissue disruption. (2) The area of exposure was vast, extending upward to expose the joint capsule and joint cavity, and downward to expose the lateral edge of the scapula. (3) The brachial plexus or axillary vessels need not be exposed. (4) The incision was relatively small and concealed. (5) As the incision is made in the axilla, the fracture can be reduced under direct vision, and the screw can be driven into the perpendicular fracture line. Additionally, the implant serves as a buttress plate by providing support to the anteroinferior region of the glenoid. (6) Exploration of the joint cavity can be facilitated by proximally flipping the fracture block, thereby removing the hematoma and any free cartilage or bone fragments, followed by examination of the damaged labrum. (7) In most instances, fracture fragments can be repositioned and stabilized extraarticularly without incising the joint capsule, thereby effectively preventing shoulder dislocation. (8) The circumflex scapular vessels can be directly visualized and protected.

However, the T-W approach has some disadvantages: (1) The incision is proximal to the axillary hair follicle, lymph nodes, and fat layer, which theoretically increases the risk of infection, necrosis, and fat liquefaction. (2) The posterior glenoid was not clearly exposed.

In conclusion, the T-W approach fills the gap in inferior glenoid fracture management, and preserves rotator cuff integrity while providing direct access to difficult fracture patterns, offering minimal invasiveness, rigid fixation, and high functional outcomes with concealed incisions. This approach is particularly valuable for all Ideberg types Ia and II, parts IV and V glenoid fractures. Surgeons should be mindful of axillary nerve anatomy during dissection. Early rehabilitation is feasible because of minimal soft tissue disruption. This study has some limitations: (1) the mean 17.8-month follow-up period may be insufficient to assess long-term outcomes; (2) the lack of a control group precludes direct comparison with established techniques; (3) a potential selection bias exists due to surgeon preference in borderline cases; and (4) the learning curve was not formally evaluated. Future studies should include longer follow-up periods, comparative trials, and standardized cumulative sum analysis to validate and refine this approach.

## Data Availability

The data that support the findings for this study are available to other researchers from the corresponding author upon reasonable request.

## Research registration unique identifying number (UIN)

Unique identifying number or registration ID: NCT05279261 (https://clinicaltrials.gov/study/NCT05279261?term=NCT05279261&rank=1).

## Assistance with the study

We would like to thank Editage (www.editage.cn) for editing the English language.

## References

[1] Armitage BM Wijdicks CA Tarkin IS. Mapping of scapular fractures with three-dimensional computed tomography. J Bone Joint Surg Am 2009;91:2222–28. 19724000 10.2106/JBJS.H.00881

[2] Cole PA Freeman G Dubin JR. Scapula fractures. Curr Rev Musculoskelet Med 2013;6:79–87. 23341034 10.1007/s12178-012-9151-x PMC3702760

[3] Judet R. Surgical treatment of scapular fractures. Acta Orthop Belg 1964;30:673–78. 14291980

[4] Obremskey WT Lyman JR. A modified judet approach to the scapula. J Orthop Trauma 2004;18:696–99. 15507823 10.1097/00005131-200411000-00007

[5] Jones CW George VM Hong TF. The inter-teres approach to glenoid neck fractures: an alternative approach to glenoid fixation. J Shoulder Elbow Surg 2018;27:1290–96. 29305097 10.1016/j.jse.2017.11.021

[6] Agha RA Mathew G Rashid R. Transparency In The reporting of Artificial INtelligence – the TITAN guideline. Premier J. Sci 2025;10:100082.

[7] van Oostveen DP Temmerman OP Burger BJ et al.. Glenoid fractures: a review of pathology, classification, treatment and results. Acta Orthop Belg 2014;80:88–98. 24873091

[8] Morshed S Corrales L Genant H Miclau T. 3^rd^ outcome assessment in clinical trials of fracture-healing. J Bone Joint Surg Am 2008;90:62–67. 10.2106/JBJS.G.01556 18292359

[9] Hudak PL Amadio PC Bombardier C. Development of an upper extremity outcome measure: the DASH (disabilities of the arm, shoulder and hand) [corrected]. The Upper Extremity Collaborative Group (UECG). Am J Ind Med 1996;29:602–08. 8773720 10.1002/(SICI)1097-0274(199606)29:6<602::AID-AJIM4>3.0.CO;2-L

[10] Constant CR Murley AH. A clinical method of functional assessment of the shoulder. Clin Orthop Relat Res 1987;214:160–64. 3791738

[11] Ideberg R Grevsten S Larsson S. Epidemiology of scapular fractures. Incidence and classification of 338 fractures. Acta Orthop Scand 1995;66:395–97. 7484114 10.3109/17453679508995571

[12] Brodsky JW Tullos HS Gartsman GM. Simplified posterior approach to the shoulder joint. A technical note. J Bone Joint Surg Am 1987;69:773–74. 3597479

[13] Wirth MA Butters KP Rockwood CA Jr. The posterior deltoid-splitting approach to the shoulder. Clin Orthop Relat Res 1993;296:92–98. 8222457

[14] Gauger EM Cole PA. Surgical technique: a minimally invasive approach to scapula neck and body fractures. Clin Orthop Relat Res 2011;469:3390–99. 21761253 10.1007/s11999-011-1970-3 PMC3210267

[15] Cole PA. Scapula fractures. Orthop Clin North Am 2002;33:1–18. 11832310 10.1016/s0030-5898(03)00069-5

[16] Scheibel M Habermeyer P. Subscapularis dysfunction following anterior surgical approaches to the shoulder. J Shoulder Elbow Surg 2008;17:671–83. 18329294 10.1016/j.jse.2007.11.005

[17] Hardegger FH Simpson LA Weber BG. The operative treatment of scapular fractures. J Bone Joint Surg Br 1984;66:725–31. 6501369 10.1302/0301-620X.66B5.6501369

[18] Scheibel M Magosch P Lichtenberg S Habermeyer P. Open reconstruction of anterior glenoid rim fractures. Knee Surg Sports Traumatol Arthrosc 2004;12:568–73. 15034646 10.1007/s00167-004-0495-7

[19] Cameron SE. Arthroscopic reduction and internal fixation of an anterior glenoid fracture. Arthroscopy 1998;14:743–46. 9788371 10.1016/s0749-8063(98)70102-1

[20] Tuman JM Bishop JA Abrams GD. Arthroscopic reduction and internal fixation of an inferior glenoid fracture with scapular extension (Ideberg V). Arthrosc Tech 2015;4:e869–72. 27284526 10.1016/j.eats.2015.08.012 PMC4886700

